# Supplementary material for: Identifying the ubiquitination targets of E6AP by orthogonal ubiquitin transfer
Source: Nat Commun. 2017 Dec 20;8:2232. doi: 10.1038/s41467-017-01974-7 (PMC5738348; doi:10.1038/s41467-017-01974-7)
Supplement: Supplementary file 1 — Supplementary Information [file 41467_2017_1974_MOESM1_ESM.pdf]

Supplementary Information

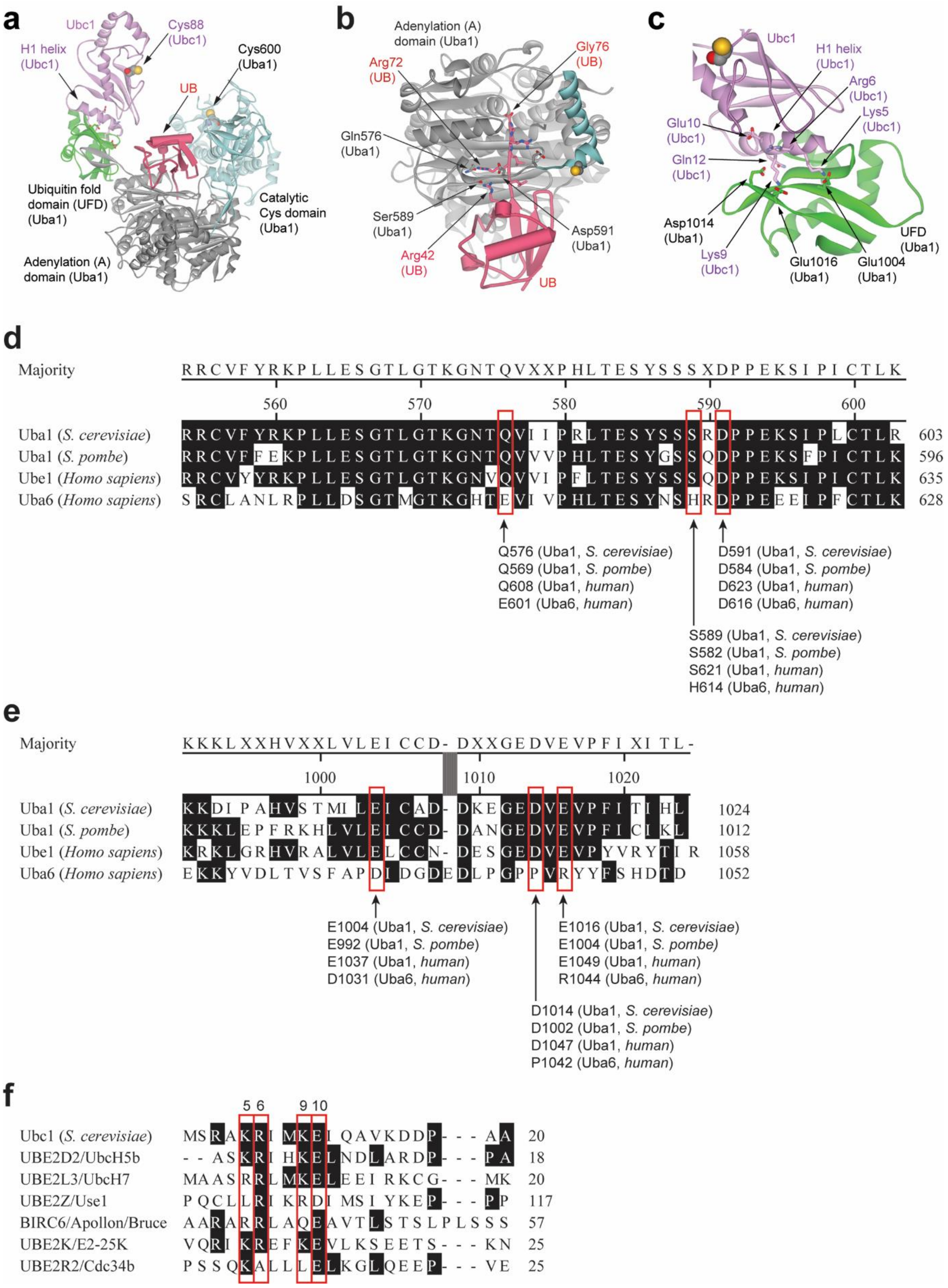

Supplementary Figure 1. Structure and sequence analysis of the UB-E1 and E1-E2 interfaces. (**a**) Modeled structures of *S cerevisiae* Uba1 (E1) in complex with UB and Ubc1 (E2). Ubc1 was modeled into the Uba1-UB complex (PDB ID 3CMM) [19]. (**b**) The binding interface between Uba1 and UB. R42E and R72E mutations were incorporated into UB to generate xUB. Mutations Q576R, S589R and D591R were incorporated into the adenylation domain of yeast Uba1 to complement the mutations in xUB and restore xUB transfer to xUba1[17]. (**c**) The binding interface between Uba1 and Ubc1. Mutations E1004K, D1014K and E1016K were incorporated into the UFD domain of yeast Uba1 with matching mutations in the Ubc1 (K5D, R6E, K9E, E10Q, Q12L) to generate the xUba1-xUbc1 pair [17]. (**d**) Regions of adenylation domains of E1s from yeast and human important for UB binding. (**e**) Regions of UFD domains of E1s important for binding to the N-terminal helices of E2s. (**f**) Sequence alignment of the N-terminal helices of the E2s from yeast and human.

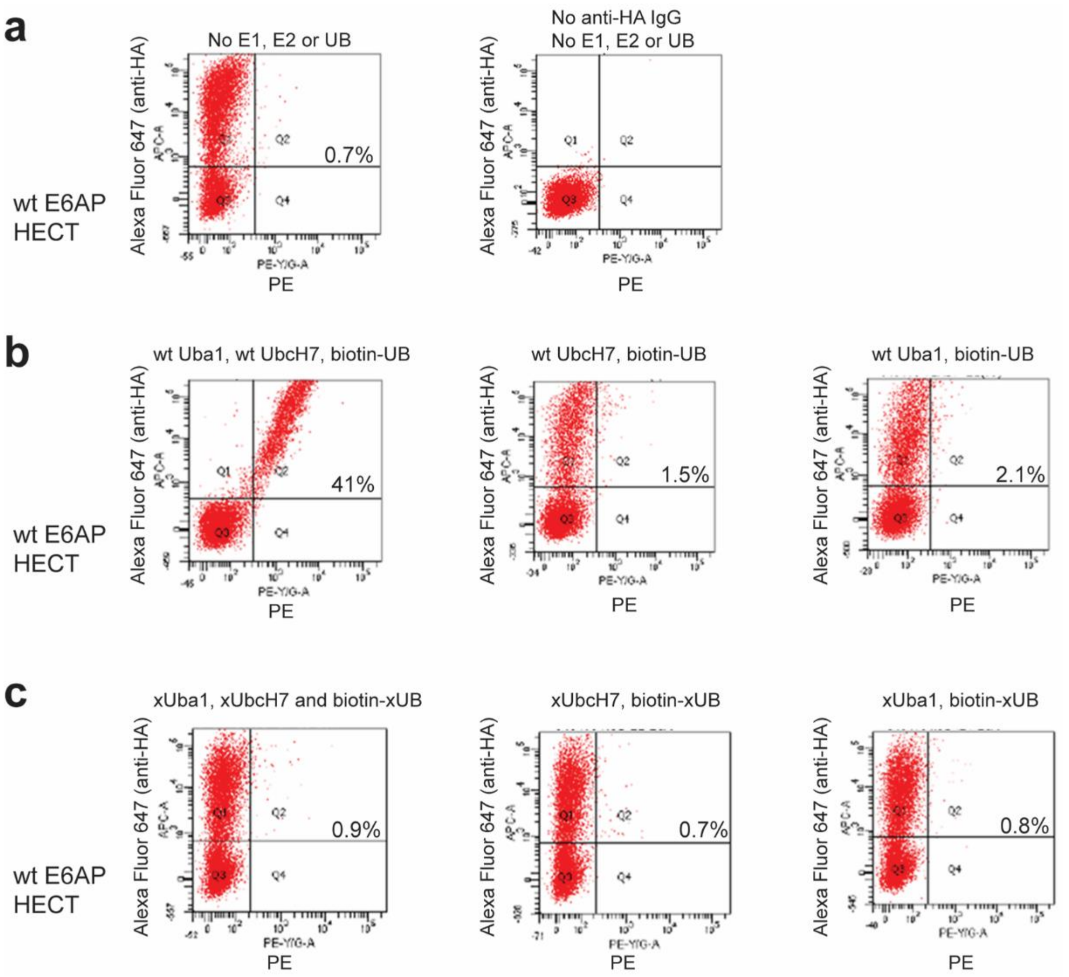

Supplementary Figure 2. Model selection of yeast cells displaying wt HECT domain of E6AP and analysis by flow cytometry. Numbers in the figure denote the percentages of cells doubly labeled with Alexa Fluor 647 and PE. (**a**) Yeast cells displaying the wt HECT were labeled with anti-HA antibody and subsequently with anti-IgG-Alexa Fluor 647 and streptavidin-PE. In the control labeling reaction (right panel), yeast cells displaying the wt E6AP HECT were directly labeled with streptavidin-PE and anti-IgG-Alexa Fluor 647 conjugates. (**b**) Yeast cells displaying the wt E6AP HECT were reacted with wt Uba1, UbcH7 and biotin-UB and bound to anti-HA antibody. Cells were labeled with anti-IgG-Alexa Fluor 647 and streptavidin-PE as secondary reagents. In the control reactions (right two panels), either wt Uba1 or wt UbcH7 was excluded in the UB transfer reaction. FACS analysis showed that a significant portion of cells (>40%) were double labeled with PE and Alexa 647 when both Uba1 and UbcH7 were added to the reaction. In contrast, when either Uba1 or UbcH7 was missing from the reaction, the cells were no longer labeled with PE, but still, a major portion of the cell (~50%) were singly labeled with Alexa 647. These results suggest that E6AP HECT displayed well on the yeast cell and was reactive with the Uba1-UbcH7 pair for UB transfer. (**c**) Yeast cells displaying the wt HECT domain were reacted with

xUba1, xUbcH7 and biotin-xUB and bound to anti-HA antibody. Cells were labeled with streptavidin-PE and anti-IgG-Alexa Fluor 647 conjugates. In the control reactions (right two panels), either xUba1 or xUbcH7 was excluded in the xUB transfer reaction. Here, the cells were no longer labeled with PE demonstrating that wt HECT could not relay with the xUba1-xUbcH7 pair for xUB loading.

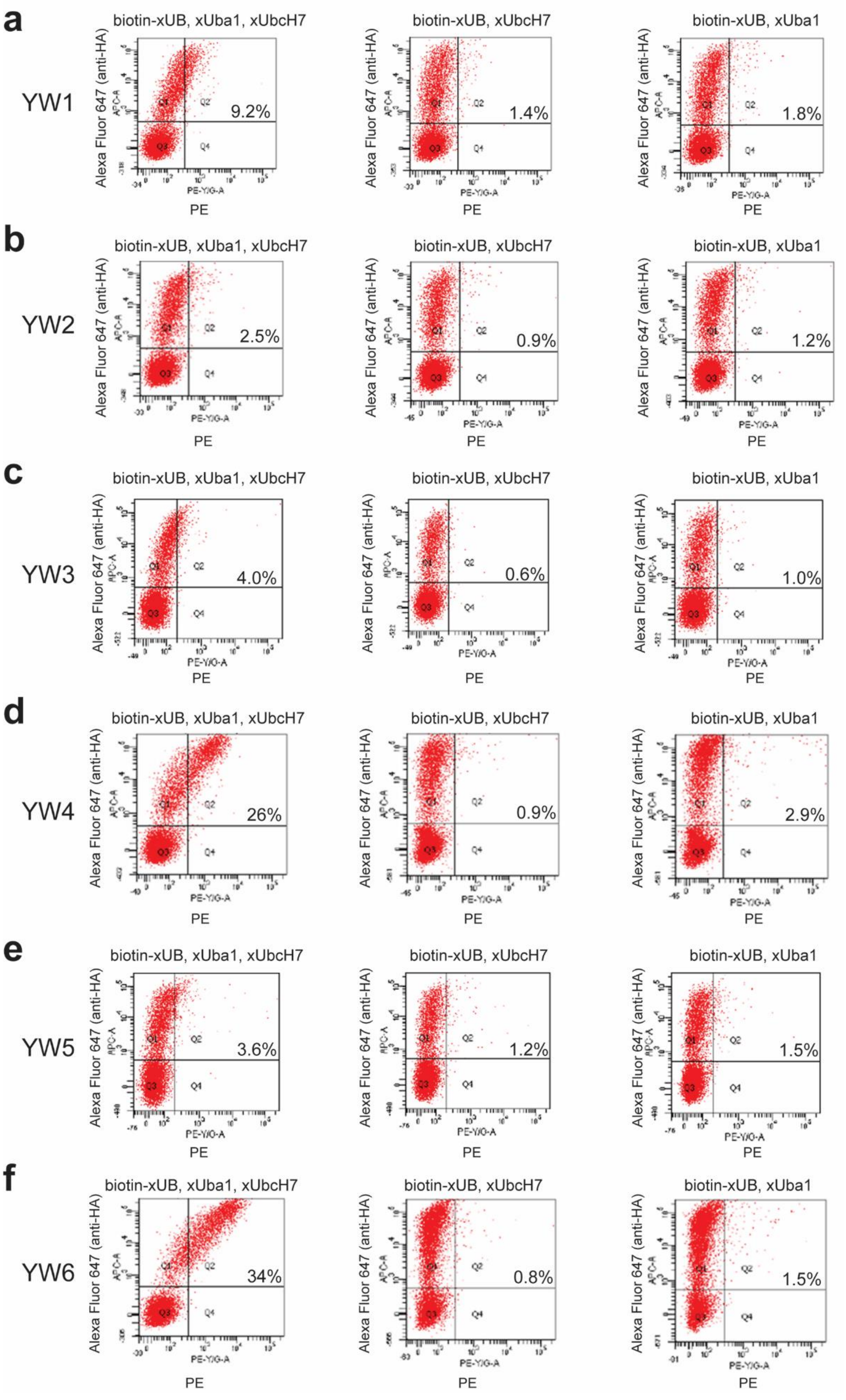

a
YW1
biotin-xUB, xUba1, xUbcH7
9.2%
biotin-xUB, xUbcH7
1.4%
biotin-xUB, xUba1
1.8%
b
YW2
biotin-xUB, xUba1, xUbcH7
2.5%
biotin-xUB, xUbcH7
0.9%
biotin-xUB, xUba1
1.2%
c
YW3
biotin-xUB, xUba1, xUbcH7
4.0%
biotin-xUB, xUbcH7
0.6%
biotin-xUB, xUba1
1.0%
d
YW4
biotin-xUB, xUba1, xUbcH7
26%
biotin-xUB, xUbcH7
0.9%
biotin-xUB, xUba1
2.9%
e
YW5
biotin-xUB, xUba1, xUbcH7
3.6%
biotin-xUB, xUbcH7
1.2%
biotin-xUB, xUba1
1.5%
f
YW6
biotin-xUB, xUba1, xUbcH7
34%
biotin-xUB, xUbcH7
0.8%
biotin-xUB, xUba1
1.5%
Alexa Fluor 647 (anti-HA)
APC-A
PE-Y/G-A
PE

Supplementary Figure 3. Verifying the activity of the selected yeast clones in HECT modification with biotin-xUB through the xUba1-xUbcH7 pair. Clones YW1-6 were reacted with biotin-xUB in the presence of xUba1 and xUbcH7. Biotin-xUB conjugated to HECT was detected by binding to streptavidin-PE and the display of HECT domain on cell surface was detected with a mouse anti-HA IgG and an anti-mouse IgG-Alexa 647 conjugate. In the control reaction (the panels on the right), either xUba1 or xUbcH7 was missing from the reaction. The percentage in each panel designates the fraction of doubly labeled yeast cells counted by flow cytometry.

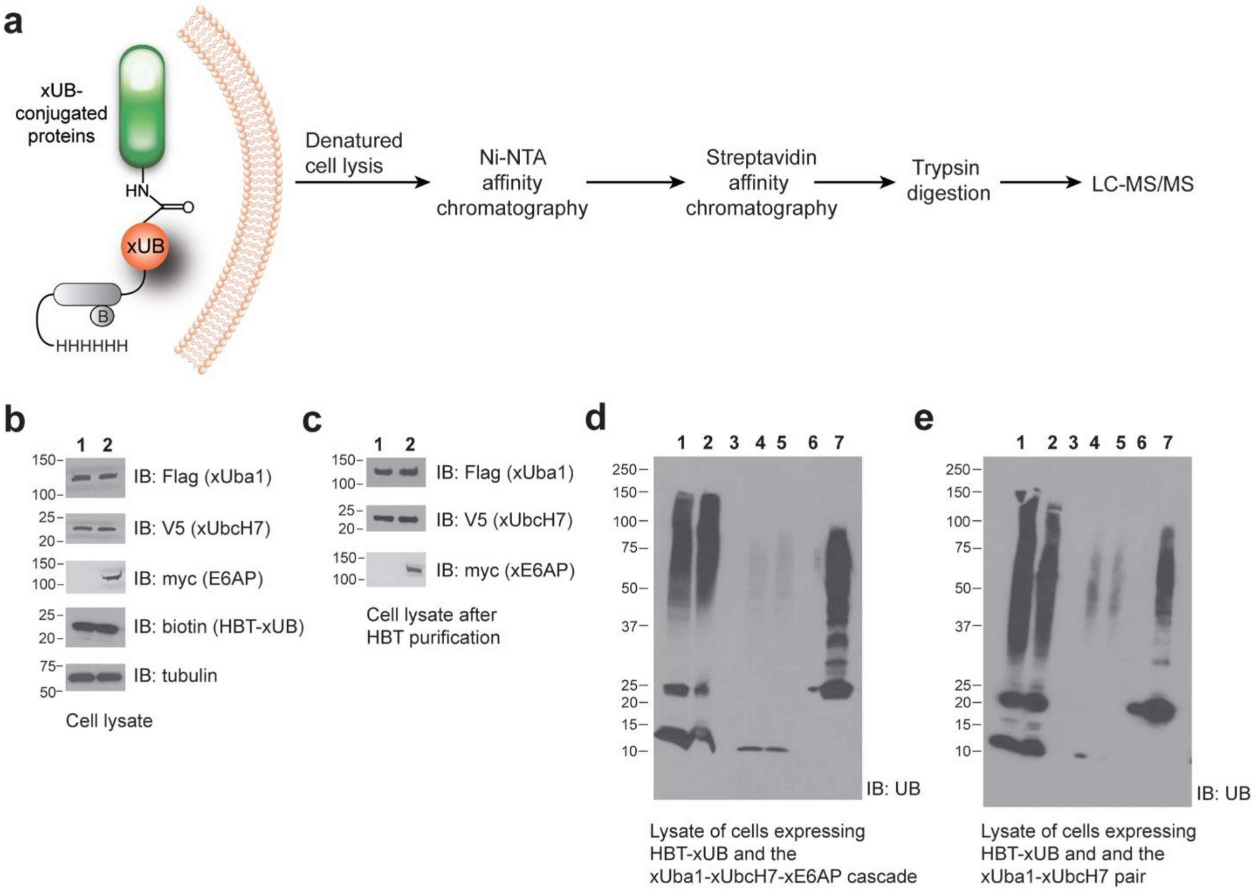

Supplementary Figure 4. Using OUT cascade to identify E6AP substrates. (a) HBT-xUB and the OUT cascade of xUba1-xUbcH7-xE6AP were expressed in the HEK293 cells. xUB-conjugated proteins in the cell were purified by sequential affinity chromatography to bind to the 6×His tag and the biotin tag on xUB. The identities of purified proteins were then revealed by tandem mass spectrometry. (**b**) Verification of the expression of HBT-xUB and the xUba1-xUbcH7-xE6AP cascade in HEK293 cells stably transfected with lentiviruses (lane 2). Expression levels of various OUT components were assayed with antibodies against the Flag, V5 and myc tags fused to xUba1, xUbcH7 and xE6AP, respectively. Expression of HBT-xUB was revealed by probing the Western blot with streptavidin-HRP. A control cell line was used for the expression of HBT-xUB and the xUba1-xUbcH7 pair (lane 1). (**c**) Verification of xUB transfer to xUba1, xUbcH7 and xE6AP in the stable cell line expressing the OUT cascade (lane 2). Proteins conjugated to HBT-xUB were purified by tandem affinity chromatography from the stable cell line. The presence of xUba1, xUbcH7 and xE6AP among the purified proteins were verified by Western blots. Stable cell line for the expression of xUba1-xUbcH7 pair was also assayed for xUB conjugation. (**d** and **e**) Progress of purification of xUB conjugated proteins through sequential affinity columns of Ni-NTA and streptavidin. Stable cell lines expressing the full-length OUT cascade xUba1-xUbcH7-xE6AP

was used in (**d**) and stable cell lines expressing truncated OUT cascade with the xUba1-xUbcH7 pair was used in (**e**). Lane 1, cell lysate; lane 2, flow-through from the Ni-NTA column; lane 3, wash of the Ni-NTA column; lane 4, elution from the Ni-NTA column; lane 5, flow-through from the streptavidin column; lane 6, wash of the streptavidin column; lane 7, protein bound to the streptavidin beads after washing. All blots are representative of at least three independent experiments.

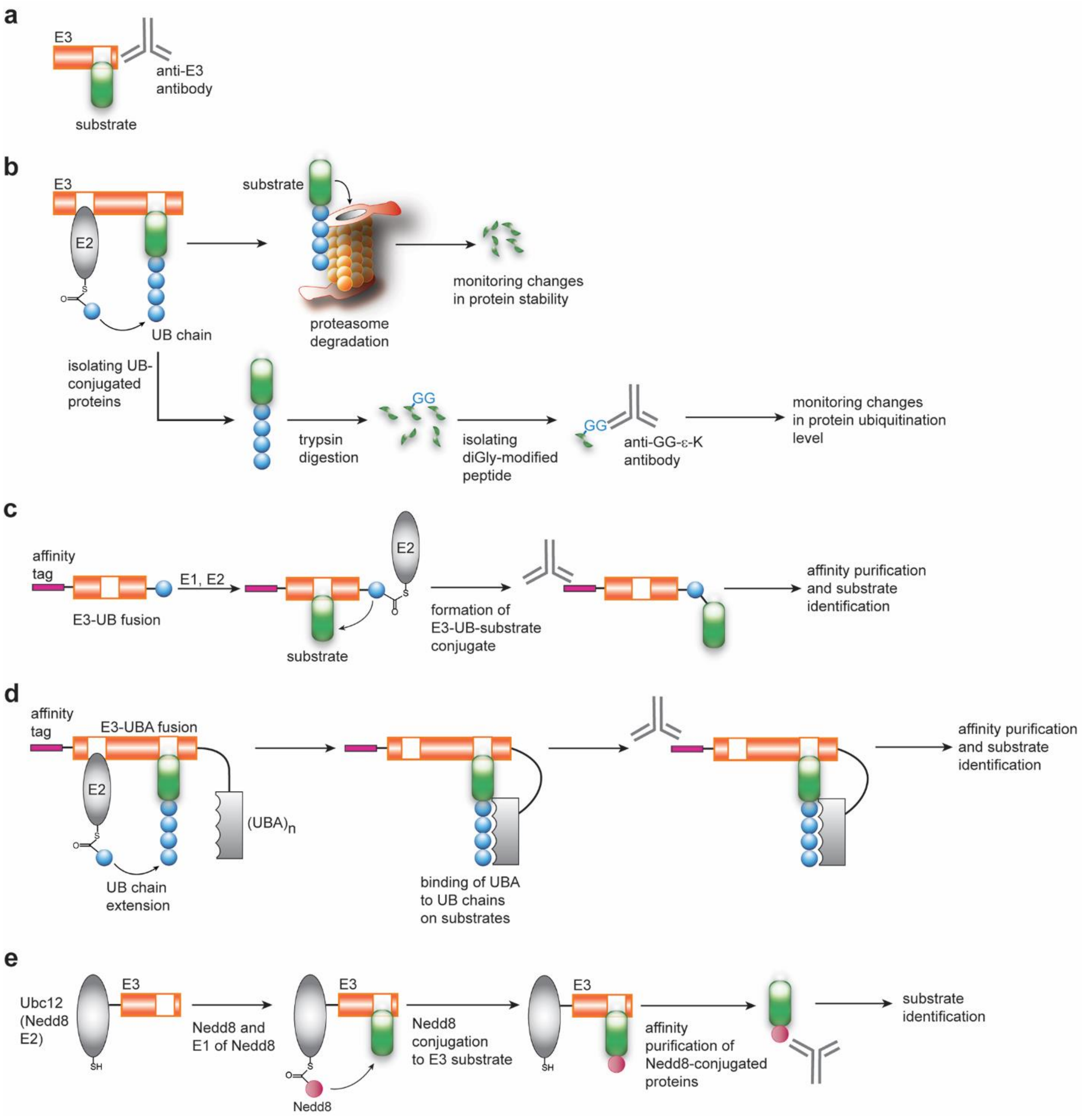

Supplementary Figure 5. Methods for identifying E3 substrates. (**a**) Identifying E3 substrates based on affinity binding between E3 and substrate proteins. (**b**) Identifying E3 substrates by monitoring changes in protein stability upon perturbation of E3 activity with a small molecule inhibitor, shRNA, or by overexpression of an E3. Alternatively, ubiquitinated proteins could be isolated from the cell and trypsin digestion would generate peptide fragments with diGly modification at Lys residues as reminiscent of UB conjugation to the substrate proteins. These fragment could be purified by an anti-GG-

ε-K antibody and their levels be monitored by MS to correlate changes in protein ubiquitination level with the up or down regulation of E3 activity. (**c**) For "UBAIT", an E3-UB fusion was used to form conjugates with E3 substrates due to the *in cis* transfer of UB to substrates bound to E3. (**d**) An E3-UBA fusion was used to bind to polyubiquitnated proteins synthesized by the same E3 enzymes. (**e**) To generate "Neddylator" for E3 substrate identification, Ubc12, the E2 for the Nedd8 protein, was fused with E3. The fusion would transfer Nedd8 to E3 substrates to facilitate their identification in the pool of Nedd8-modified proteins in the cell.

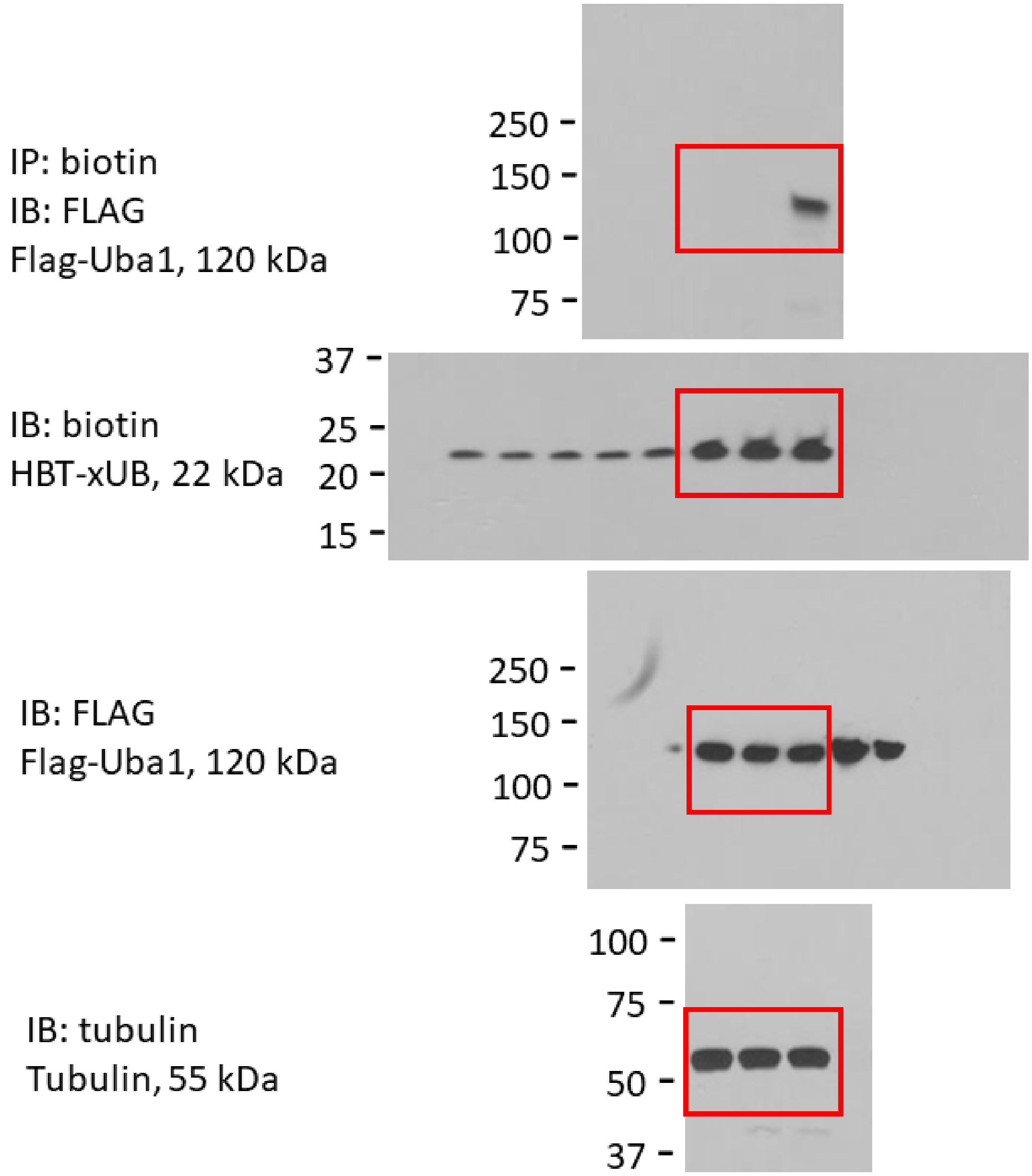

Supplementary Figure 6. Uncropped Western blots in Fig. 4b.

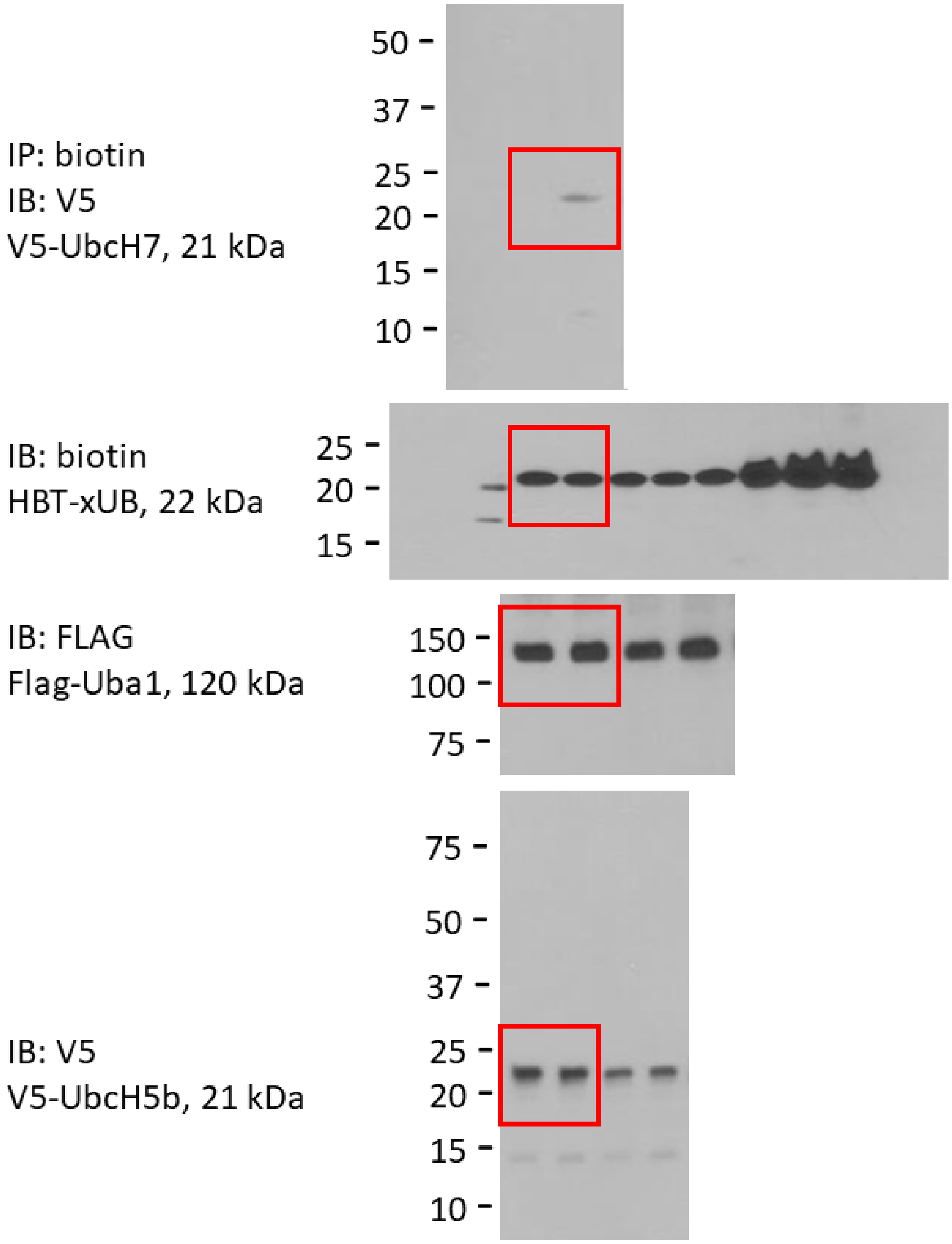

Supplementary Figure 7. Uncropped Western blots in Fig. 4c.

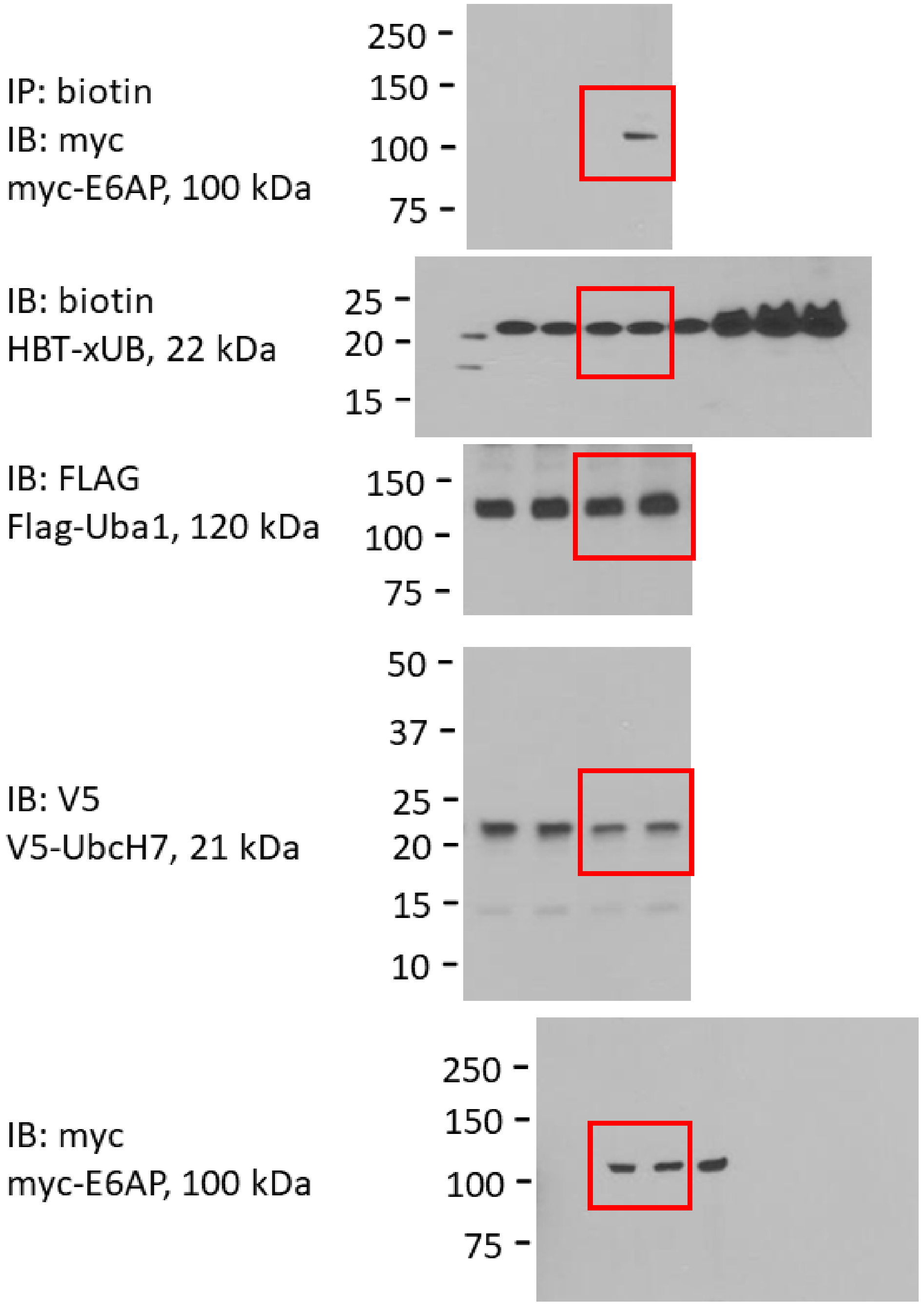

Supplementary Figure 8. Uncropped Western blots in Fig. 4d.

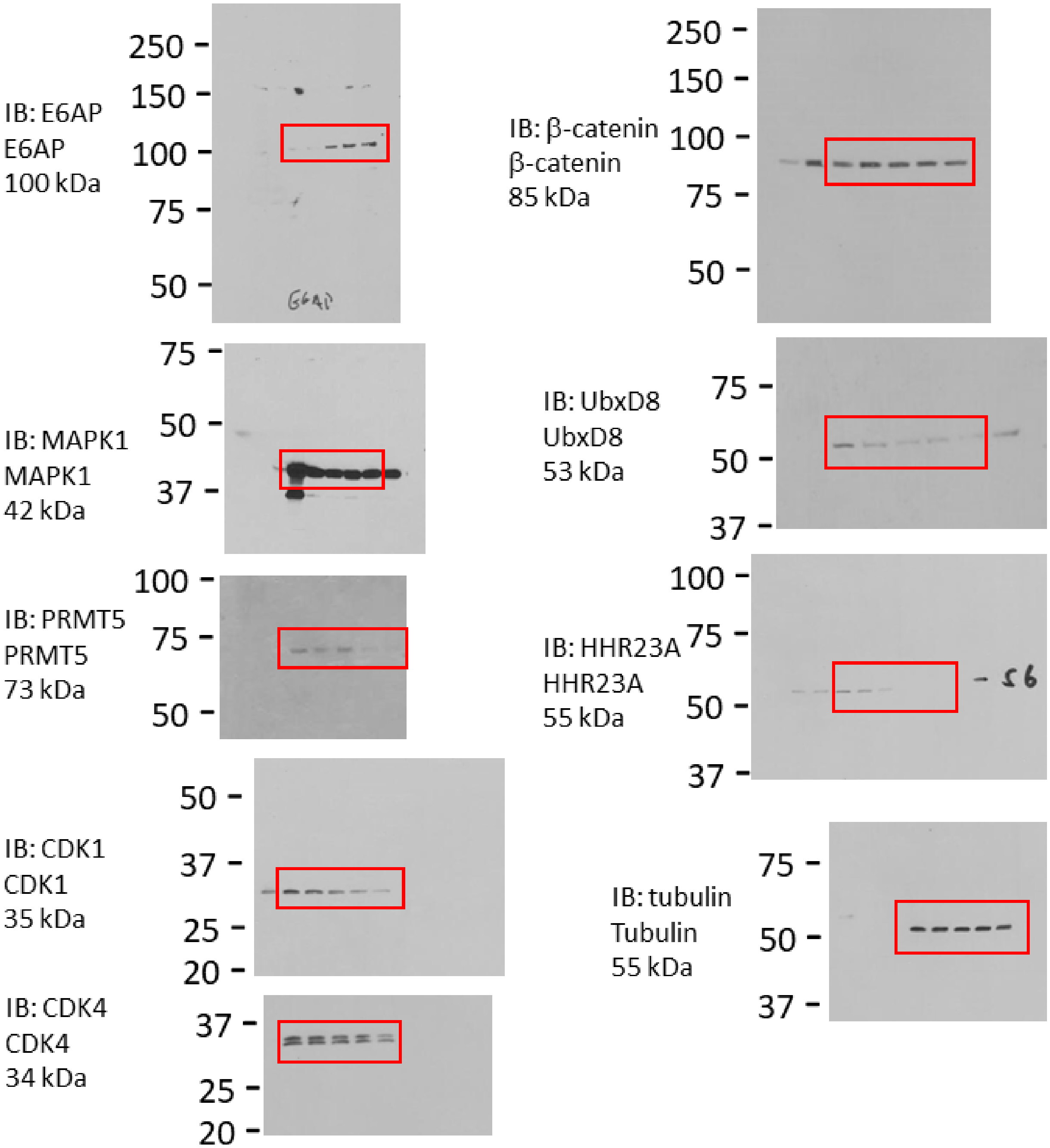

Supplementary Figure 9. Uncropped Western blots in Fig. 7a.

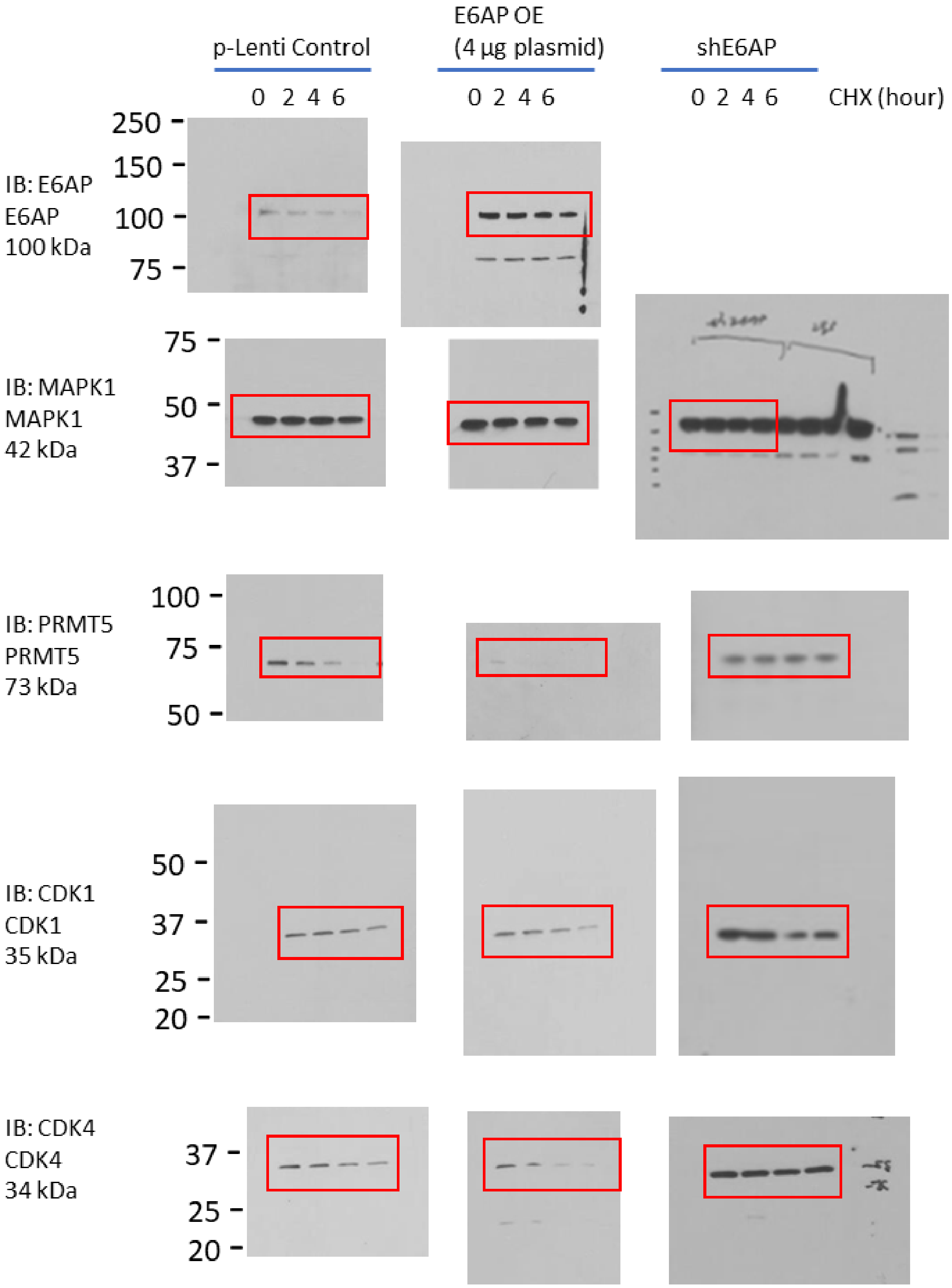

Supplementary Figure 10. Uncropped Western blots in Fig. 7c (part 1).

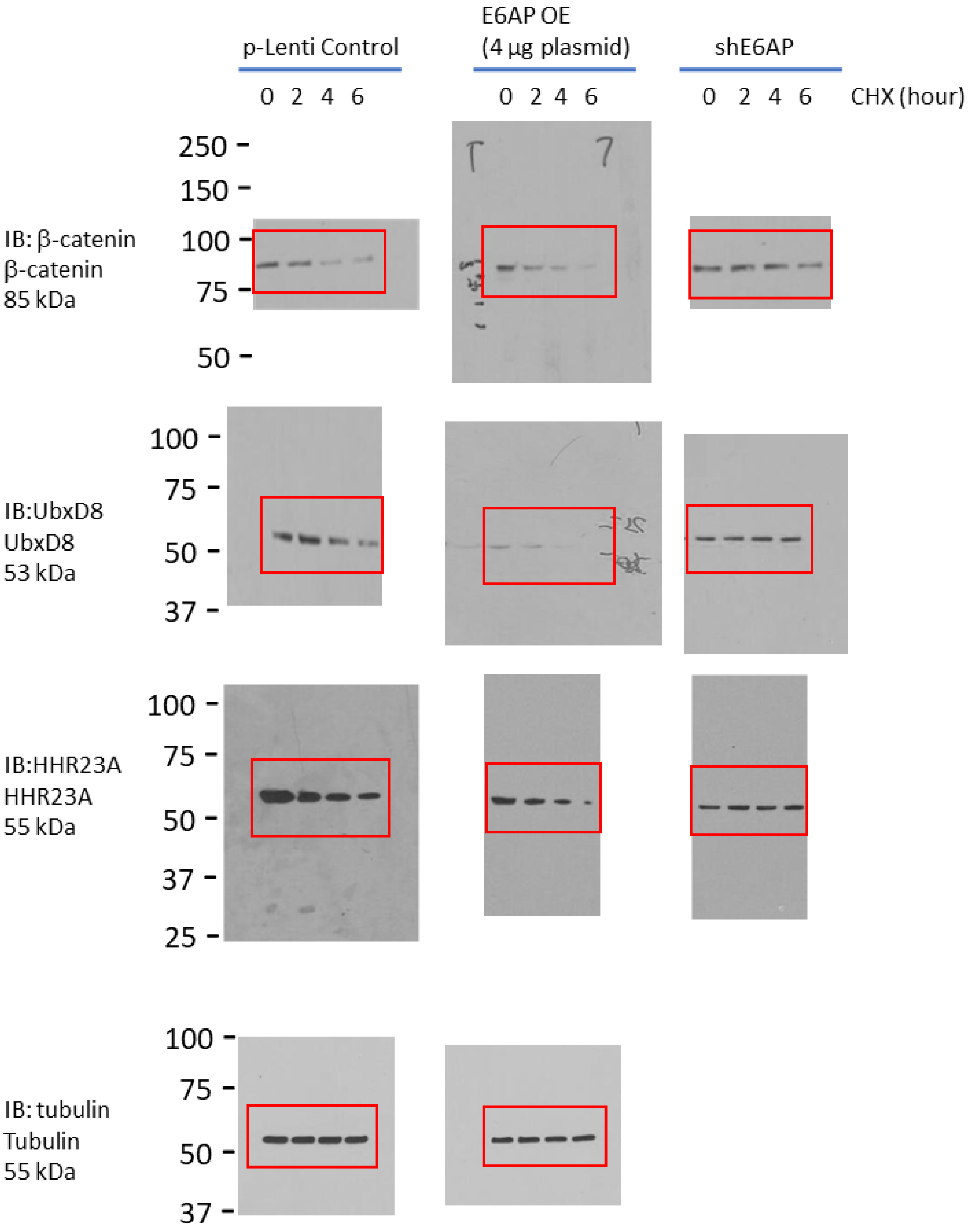

Supplementary Figure 11. Uncropped Western blots in Fig. 7c (part 2).

Supplementary Table 1. Sequences of the primers used in this study.

Primer	Sequence
WY 1	5’-GACAAGCCGCGGGAAAGAGATCTAGTCCAGAAGCTGAAAGTC-3’
WY 2	5’-GACAAGCCGCGGAAGCGAGACCTGGTTCAGAAACTAAAAATTTTGCGG-3’
WY 3	5’-GACAAGCCGCGGGAATTTAAGCAGAAATATGACTACTTCAGG-3’
WY 4	5’-GACAAGCCGCGGCAGTTGAATCCATATTTGAGAC-3’
WY 5	5’-CTCGAGTGCGGCCGCCTACTCCACAGCAAACCCGCAGGTCTC-3’
WY 6	5’-CTCGAGTGCGGCCGCCTATTCCACAGCAAATCCACATGTTTC-3’
WY 7	5’-CATCTT CTCGAGTGCGGCCGCCTAATCCACCCCTTCAAATCC-3’
WY 8	5’-AGGCATGCTTGCGGCCGCCTACAGCATGCCAAATCCTTTGG-3’
WY 9	5’-GATATACATATGGAGCTCATGGCGGCCAGCGAGAGGCTGATGGAGGAG CTTGAAGAAATC -3’
WY 10	5’-GATATACTCGAGACTAGTGGTACCGTCCACAGG-3’
WY 11	5’-GGGTCGGCTAGCCAGTTGAATCCATATTTGAGACTC-3’
WY 12	5’-CTGATGCTCGAGCTACAGCATGCCAAATCC-3’
WY 13	5’-TTCCACATTCCCTTC-3’
WY 14	5’-GAAGGGAATGTGGAANNKNNKNNKNNKATCNNKTTCCAGATATCACAGAC-3’
WY 15	5’-ACTGCTGAATTCACCCCCAAGTCTGGCGTCAAG-3’
WY 16	5’-GGCTAGTGGCGCGCCTCATCAGCGGATGGTGTATCG-3’
WY 17	5’-ACCATGAGCGCTATGGCGGCCAGCGAGAGGCTGATGGAGGAGC-3’
WY 18	5’-ACCCTTGCTAGCGTCCACAGGTCGCTTTTCC-3’
WY 19	5’-GATCTCGCTAGCATGAAGCGAGCAGCTGCAAAGCATCTAATAGAACGC-3’
WY 20	5’-GATCTCGGGCCCTCACAGCATGCCAAATCCTTTGGCATACG-3’
WY 21	5’-GACAAGCCGCGGGCTGCAAAGCATCTAATAGAACGCTACTACCATCAGTTAACTGA GGGCTGTGGAAATGAG-3’
KB 1	5’-GGGATCCCATATGGAAGATTATACCAAAATAGAGAAAATTGG -3’
KB 2	5’-TAATCTGGCACCTCGAGCATCTTCTTAATCTGATTGTCC -3’
KB 3	5’- GATATCGAGTACTATGGCTGCCACTCGATATGAACCCG-3’
KB 4	5’- CCCGGGCTGCGGCCGCTCACTCTGCGTCGCTTTCCTCCTTGTGCAGG-3’
Bo13	5’-GTACTTGACACAACCG-3’
Bo73	5’-CGGAGCTCGAATTCTCATAGATGAATGGTAATGAAAGGAACTTTAACCTTCTCTCCT TCCTTGTCAT CTGCGCAAATTTTGAGAATCATTGTAGATAC-3’
Bo184	5’-CGAGTGGTGATCCCCTTCCTGACAGAGTCGTACAGTTCCCGCCAGCGCCCACCT GAGAAGTCCATCCCC-3’
Bo185	5’-CGACTCTGTCAGGAAGGGGATCACCACTCGCACATTGCCTTTGGTGCCCAG-3’
Bo186	5’-GCTCAGCATGGCCGGCCACC-3’
Bo187	5’-GCCAGACTTGGGGGTGAATTC-3’
Bo188	5’-GCTCGAGTGCGGCCGCTCAGCGGATGGTGTATCGGACATAGGGAACCTTGAC CTTCTCGCCGCTCTCGTCGTTACAGCACAGCTTAAGCACCAGCGCCCGCAC-3’
LZ1	5’-ATATATCATATGATGGCGGCGCCTGAGG-3’
LZ2	5’-TGCTCGAGCGGCCGCTCATTCGTCAGTTAGGTC-3’
