# Supplementary material for: Identifying the ubiquitination targets of E6AP by orthogonal ubiquitin transfer
Source: Nat Commun. 2017 Dec 20;8:2232. doi: 10.1038/s41467-017-01974-7 (PMC5738348; doi:10.1038/s41467-017-01974-7)
Supplement: Supplementary file 3 — Description of Additional Supplementary Files [file 41467_2017_1974_MOESM3_ESM.pdf]

Description of Additional Supplementary Files

File Name: Supplementary Data 1
Description: **Potential E6AP substrates identified by orthogonal UB transfer (OUT).** Numbers of peptide-spectrum match (PSM) for xUB-conjugated proteins purified from cells expressing the full length OUT cascade of E6AP (xUba1-xUbcH7-xE6AP) are listed under "OUT Screen 1", "OUT Screen 2" and "OUT Screen 3", respectively. Numbers of PSM for xUB conjugated proteins purified from cells expressing the truncated OUT cascade (xUba1-xUbcH7) without xE6AP are listed under "Control 1", "Control 2" and "Control 3", respectively. The OUT screens were repeated three times, each time with different preparation of the cells. Proteins appearing in all three biological replicates with PSM ratios greater than 2 (Log2 of the PSM ratios greater than 1) are listed in the table. PSM ratios are calculated as the Log2 of the ratios of the PSM of the protein from the OUT screen cells to the same protein from the control cells. Average of the PSM ratios of the three biological replicates are listed in the last column. Literature reported E6AP substrates identified in the list are colored in an orange background. .E6AP substrates MAPK1, PRMT5, CDK1, CDK4, β-catenin, and UbxD8 verified in this study are colored in a green background.

File Name: Supplementary Data 2
Description: **Ingenuity canonical pathways associated significantly with E6AP substrates.**

File Name: Supplementary Data 3
Description: **Protein networks associated significantly with E6AP substrates.**

File Name: Supplementary Data 4
Description: **Analysis of potential E6AP targets based on their frequencies of appearance in the CRAPome database.** Known E6AP targets are colored with an orange background. Verified E6AP targets in this study are colored with a green background. All targets are sorted based on their frequencies in the CRAPome database (www.crapome.org).

File Name: Supplementary Data 5
Description: **Proteins appearing only in the control samples of the OUT screen.** During the OUT screen, control experiments were set up with the expression of HBT-xUB and the xUba1-xUbcH7 cascade in the HEK293 cells without the expression of xE6AP.
After tandem purification, cellular proteins conjugated with HBT-xUB were identified in control cells and in cells expressing the full xUba1-xUbcH7-xE6AP cascade (OUT screen cell). Proteins reproducibly appearing in control cells in three biological repeats but not in any of the OUT screen cells expressing the full xE6AP cascade were listed. PSM, numbers of peptide-spectrum match.
